# Projected habitat preferences of commercial fish under different scenarios of climate change

**DOI:** 10.1038/s41598-024-61008-3

**Published:** 2024-05-03

**Authors:** Sana Sharifian, Mohammad Seddiq Mortazavi, Seyedeh Laili Mohebbi Nozar

**Affiliations:** Agricultural Research Education and Extension Organization (AREEO), Persian Gulf and Oman Sea Ecological Research Center, Iranian Fisheries Sciences Research Institute, Bandar Abbas, Hormozgan Iran

**Keywords:** Habitat suitability, Predictive model of MaxEnt, Temperature, Distributional shifts, Ecology, Climate-change ecology, Ecological modelling

## Abstract

The challenges of commercial species with the threats of climate change make it necessary to predict the changes in the distributional shifts and habitat preferences of the species under possible future scenarios. We aim to demonstrate how future climatic changes will affect the habitat suitability of three species of commercial fish using the predictive technique MaxEnt. The dataset used to extract geographical records included OBIS (54%), GBIF (1%), and literature (45%). The output of the model indicated accurate projections of MaxEnt (AUC above 0.9). Temperature was the main descriptor responsible for the main effects on the distribution of commercial fish. With increasing RCP from 2.5 to 8.5, the species would prefer saltier, higher temperatures and deeper waters in the future. We observed different percentages of suitable habitats between species during RCPs showing distinct sensitivity of each fish in facing climate changes. Negative effects from climate change on the distribution patterns of commercial fish were predicted to lead to varying degrees of reduction and changes of suitable habitats and movement of species towards higher latitudes. The finding emphasizes to implement adaptive management measures to preserve the stocks of these commercial fish considering that the intensification of the effects of climate change on subtropical areas and overexploited species is predicted.

## Introduction

The marine ecosystem is predicted to face an unknown challenge of climate change in the future^1–4^. Marine taxa response to climate changes through pole-ward and depth shifts^5,6^. It was projected the average global ocean temperature will increase up to 2 °C by the end of this century that depending on different scenarios of greenhouse gas concentrations^7^. Representative Concentration Pathways (RCPs) represent alternative greenhouse gas concentration routes (2.6, 4.5, 6.0, 8.5 W/m^2^ by 2100). In RCP 2.6, an initial increase in temperature until 2020 followed by a decline. RCP4.5 and RCP6.0 are known as two intermediate scenarios and finally, in RCP 8.5, a very marked temperature increase is predicted throughout the twenty-first century^7^. Greenhouse gas emissions have a main role in future changes of environmental factors of oceans such as temperature, oxygen content, and primary productivity affecting on shifting of the distribution of species and ecosystems^8,9^ and indirectly on the phenology and physiology of some marine species^10,11^, seafood contamination^12^, marine biodiversity and fisheries^11,13^. However, forecasting the magnitude of such impacts is challenging since responses to climate change are species-specific so that some can adapt to new conditions and others (such as the tropical marine fauna) shift distributions to find more suitable environments and to maintain a physiological optimum^14–17^.

Our study species including *Acanthopagrus latus, Planiliza klunzingeri,* and *Pomadasys kaakan* are high-consumption commercial fishes with high catch in the Persian Gulf^18^. Individually, *A. latus* as a fish inhabiting warm shallow and coastal waters experiences habitat changes during its life cycle^19,20^ and showed an increasing trend of catch from 2731 to 5410.50 tons in Iranian waters from 2011 to 2013^20,21^. *P. klunzingeri* is known as one of the commercially important dominant fish of multispecies fishery with different catch rates between the regions of the Persian Gulf^22–24^. Finally, the stock assessment of *P. kaakan* shows its high exploitation rate and the need for decreasing the fishing pressure in the Persian Gulf and Oman Sea^25^. Officially, around 80–85 million tons per year of global marine fisheries landings have been estimated since 1990 with mean annual gross revenues of around USD 100 billion^26^. During the last 60 years, an increasing trend in seafood consumption has been observed, so fish is reported to provide 20 percent of the animal protein needs of 2.9 billion people^27^. Livelihoods of between 660 and 820 million people, directly or indirectly are related to the global fisheries sector^27^. This is especially noticeable in developing countries where people depend on marine resources for food and income and has led to the threat of aquatic resources, such as the Persian Gulf where most of its stocks are under overfishing^28^ because of the catching of juvenile species and illegal fishing^29–31^. It seems that considering the stock status of these commercial fish and the sensitivity to climate change^32^, it is necessary to identify the habitat preferences and to predict distribution changes of these fish reliability under future climate scenarios in the direction of effective conservation of species and to implement sustainable fishing management strategies^16,33^. Climate change is considered an important challenge that will significantly reorganize the future of global fisheries in the long run^34^ and it is accompanied by potential economic impacts^35–37^. Locally^38^, suggested a high rate of extinction of commercial fish, as well as reduced future catch potential in the Persian Gulf under an 8.5 scenario by 2090. Any small temperature changes can affect marine organisms and related marine capture fisheries^1,39,40^. It was expected that climate change would impact maximum catch potential (MCP) through changes in species composition, with predicted increases of MCP in high latitudinal regions and decreases in the tropics^9^.

Habitat suitability (HS) and species distribution modeling (SDM) are suitable tools to predict the habitat preference of one species based on observed relationships of species occurrence records with environmental conditions^33,41,42^. These models can predict the possible effects of plausible climate change scenarios on the distribution of marine species through the identification of key habitat variables (Briscoe et al., 2019). The advantages of SDM are the ability to produce long-term, large-scale, and comparable future projections for reliable management and conservation perspectives^43–45^. MaxEnt as one of the species distribution modeling techniques has attracted a lot of attention in recent years to model the distribution of marine species under future scenarios. MaxEnt modeling algorithms select the best environmental predictor determining species distribution by assigning relative contributions by weighting the variables throughout the analysis^46,47^. MaxEnt finds the probability distribution of maximum entropy using the environmental covariates at species presence and background points and finally predicts the distribution using environmental variables at species presence^48^. This modeling technique well performs when handling presence-only data and small sample sizes^49–51^.

Here, we assess the global future distribution of commercial fish *A. latus, P.klunzingeri,* and* P. kaakan* under different global warming scenarios (RCP 2.6, RCP 4.5, RCP 6.0, and RCP 8.5) by biogeographic distribution records of species and a set of four environmental variables including mean depth, temperature, salinity, and current velocity (For future period: 2090 to 2100) to predict future distributions and habitat preferences of commercial fish, to understand how environmental variables shape the spatial–temporal patterns of commercial fish, and find out which variable will be more effective in predicting suitable environments of commercial fish under different climate change scenarios. We used different scenarios of climate change and global data of distribution in commercial fish to consider different levels of exposure related to different regions to obtain specific degrees of vulnerability.

## Results

### MaxEnt performance

In Table 1, MaxEnt outputs of the future model under each RCP are provided. Higher iterations express a larger convergence of the model for each species (Table 1). The future Model showed values of training AUC above 0.99 in all species presenting the high predictive power of MaxEnt to predict the actual distribution of these commercial fishes (Table 1). Values below the Minimum presence threshold (MPT) show unsuitable habitats for commercial fishes (Table 1).

**Table 1:** The maxEnt output of the future modeling under four RCPs from RCP 2.6 to RCP 8.5 for each species.

Species		Iterations	Training samples	Test samples	Training AUC ± SD	MPT
*A. latus*	RCP 2.6 ↓ RCP 8.5	1000	145	48	0.9932 ± 0.0009	0.0002
		1000	145	48	0.9923 ± 0.0057	0.0001
		1000	145	48	0.9938 ± 0.0053	0.0002
		1000	145	48	0.9897 ± 0.0018	0.0001
*P. klunzingeri*	RCP 2.6 ↓ RCP 8.5	960	75	25	0.9917 ± 0.033	0.0001
		860	75	25	0.9893 ± 0.0006	0.0003
		800	75	25	0.9818 ± 0.0028	0.0003
		900	75	25	0.993 ± 0.0093	0.0003
*P. kaakan*	RCP 2.6 ↓ RCP 2.6	740	349	116	0.9943 ± 0.0009	0.0004
		820	349	116	0.9941 ± 0.0005	0.0005
		1000	349	116	0.994 ± 0.0005	0.0014
		940	349	116	0.9944 ± 0.0013	0.0001

*SD* Standard Deviation, *MPT* Minimum presence threshold.

### The relative contribution of environmental predictors

**Figure 1:**
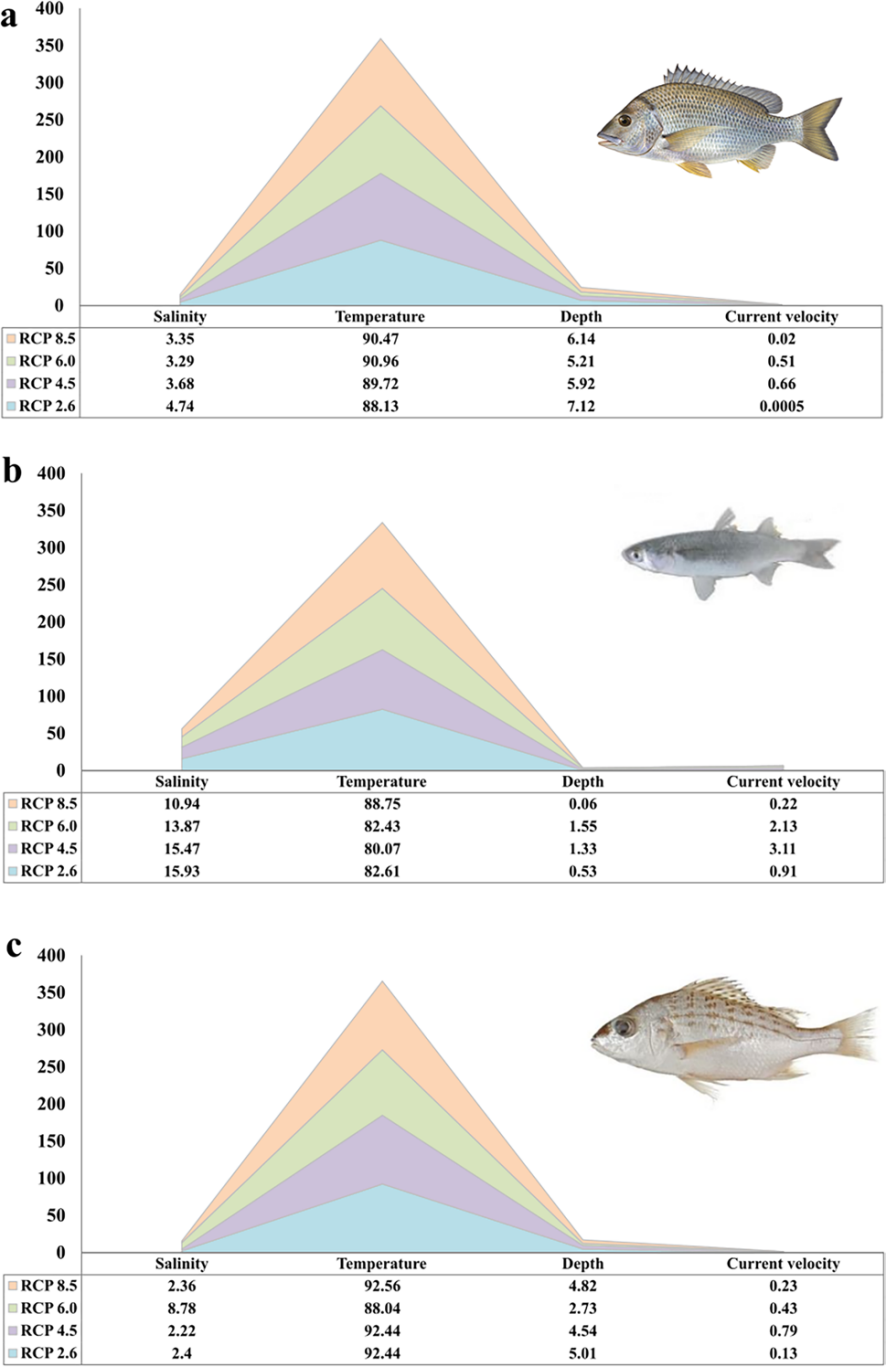
The relative importance of environmental variables to predict future distributions in three species of commercial fish including (**a**) *A. latus*, (**b**) *P. klunzingeri*, and (**c**) *P. kaakan* under different RCP scenarios.

The output of the relative contribution of each variable under four RCPs for three species is presented in Fig. 1. These values show the relative importance of environmental predictors including depth, temperature, salinity, and current velocity in predicting the future distribution of the three species under each RCP (Fig. 1). In general, the values of relative contribution showed that temperature is the strongest environmental predictors in showing the future distribution of commercial fish under four RCPs (Fig. 1). Following temperature, depth was a second dominant predictor for future distribution of fish *A. latus* and *P. kaakan* (Fig. 1). For *P. klunzingeri*, salinity was the most important variable after temperature for predicting species distribution under future scenarios (Fig. 1). Outputs of Jackknife of AUC for three species also indicated the prominent role of temperature in predicting distribution of commercial fish under future scenarios (Fig. s1).

Table 2 shows the outputs of response curves in the MaxEnt model. We observed the significant relationships between environmental variables (Spearman’s test; *P* < 0.05). In the future model, the most suitable habitats were in areas with a depth of 8.12–60.53 m, Temperature of 26.17–31.75 °C, salinity of 33.36–40.92 PSS, and currents velocity of 0.001–1.23 m^−1^ (Table 2). According to Table 2, as the scenario changes from RCP 2.6 towards RCP 8.5, the species would prefer saltier, higher temperatures and deeper waters (Table 2).

**Table 2:** Response curve output showing where there is the highest probability of predicted occurrence for three species of commercial fish under four scenarios from RCP 2.6 to RCP 8.5.

Species		Environmental predictors			
		Currents velocity	Salinity	temperature	Depth
*A. latus*	RCP 2.6 ↓ RCP 8.5	1.23	33.36	26.17	37.75
		0.001	39.18	27.75	8.12
		0.001	39.30	27.18	18.03
		1.18	39.85	28.32	60.53
*P. klunzingeri*	RCP 2.6 ↓ RCP 8.5	0.001	39.03	29.54	37.75
		0.044	39.18	30.20	35.32
		0.27	39.30	30.50	44.20
		0.038	39.25	31.75	32.71
*P. kaakan*	RCP 2.6 ↓ RCP 8.5	0.08	34.59	25.21	37.75
		0.001	34.15	27.01	8.12
		0.001	40.92	26.85	18.03
		0.092	40.63	27.03	60.53

The polynomial curves with five polynomial orders were also plotted to show the non-linear relation between temperature and salinity in latitude 5° (Fig. s2). Polynomial curves showed a significant nonlinear relationship between temperature and salinity in *P. klunzingeri* under all future scenarios (Fig. s2). The highest and lowest correlation between temperature and salinity were observed in *P. klunzingeri* and *P. kaakan* under RCP 2.6 and 8.5 scenarios, respectively (Fig. s2).

### The habitat suitability and environmental variables

**Figure 2:**
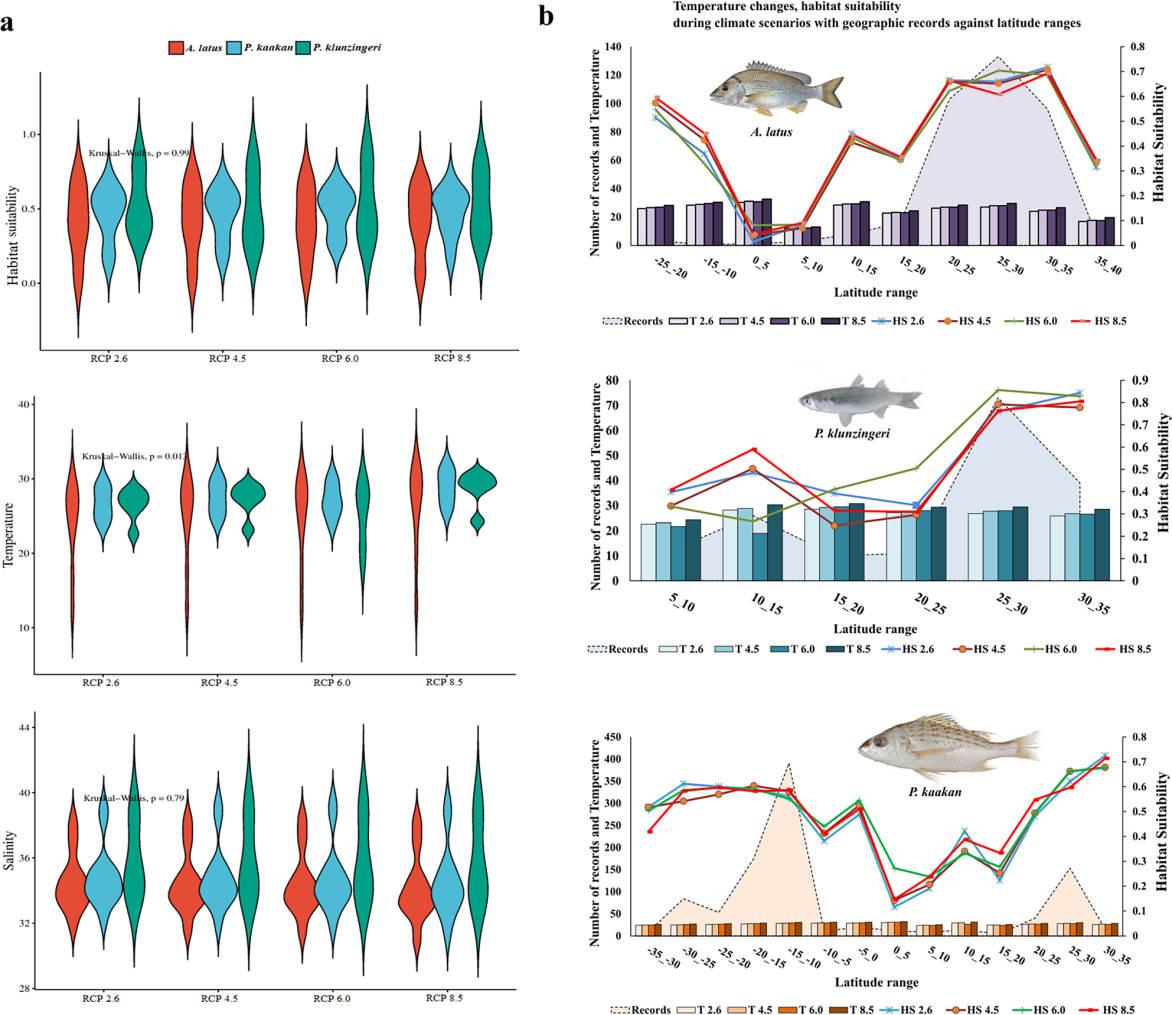
(**a**) Violin plots showing variation ranges of habitat suitability, temperature, and salinity under four RCPs in three species, and (**b**) The observed records of commercial fishes and temperature (first axis), as well as habitat suitability (second axis) versus 5° latitudinal ranges under future climate change scenarios.

Violin plots of Fig. 2a show the range of habitat suitability, temperature, and salinity under future RCPs. The median of habitat suitability was variable from 0.388 (*A. latus* in RCP 6.0) to 0.529 (*P. kaakan* in RCP 8.5) (Fig. 2a). Temperature showed significant variations between different RCPs in three species (*p* = 0.013; Fig. 2a). The minimum (26.14 °C) and maximum (29.38 °C) of median temperature were observed in *A.latus* (RCP 2.6) and *P. klunzingeri* (RCP 8.5) (Fig. 2a). The lowest (33.51 PSS) and highest (34.87 PSS) of salinity was for fish *A.latus* (RCP 8.5) and *P. klunzingeri* (RCP 2.6), respectively (Fig. 2a). The maximum number of distributional records of commercial fish )At latitude 25°-30° N for two species *A. latus* and *P. klunzingeri* and 10–15° S for *P. kaakan*) showed a high correlation with habitat suitability, so the peak of habitat preferences and high probability of occurrence of species were obtained by increasing the number of geographical records (Fig. 2b). Moreover, the temperature variations of the future scenarios along the latitudes indicated that temperature has a significant role in predicting the occurrence of species, as well as in shaping the pattern observed of geographic records of commercial fish (Fig. 2b).

### Classification and spatial distribution of habitat suitability

**Figure 3:**
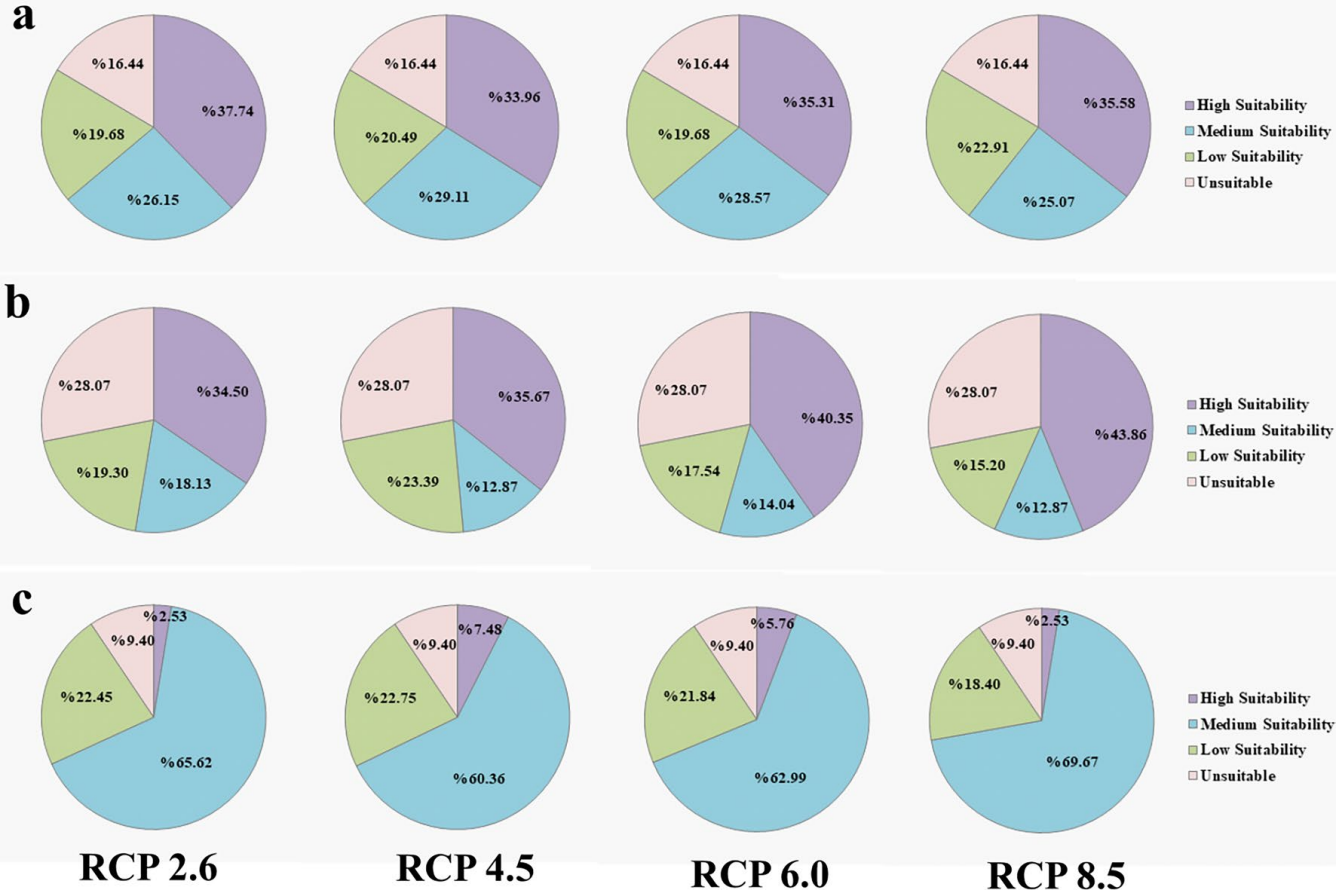
Habitat suitability predicted for three species (**a**) *A. latus*, (**b**) *P. klunzingeri*, and (**c**) *P. kaakan* during RCPs 2.6–8.5.

According to the future model of habitat suitability, fish *A. latus* and *P. klunzingeri* had a higher percentage of environments with high suitability compared to species *P. kaakan* (Fig. 3). In contrast, fish *P. kaakan* showed a much higher percentage of environments with medium suitability than the other two species (Fig. 3). Under four RCPs, maximum (46.83%) and minimum (2.53%) habitats with high suitability were belonged to fish *P. klunzingeri* and *P. kaakan*, respectively (Fig. 3). The highest (69.67%) and the lowest (12.87%) percentages of environments with medium suitability were obtained for *P. kaakan* and *P. klunzingeri*, respectively (Fig. 3). In terms of the percentage of environments with unsuitable conditions, the order of *P. klunzingeri* (28.07%), *A. latus* (16.44%) and *P. kaakan* (9.40%) was observed (Fig. 3).

**Figure 4:**
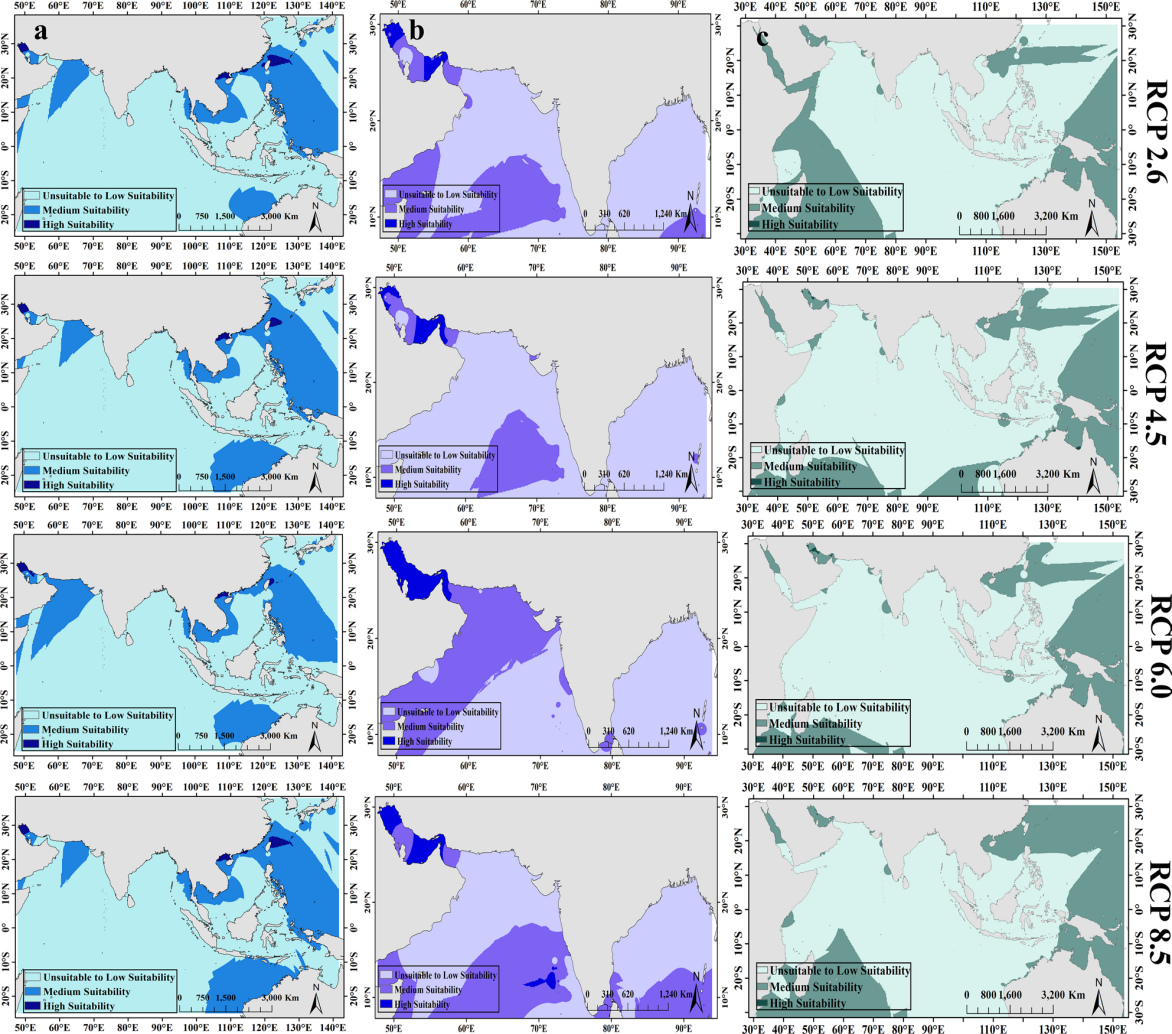
The spatial distribution of habitat suitability in projections produced for future models in three species (**a**) *A. latus*, (**b**) *P. klunzingeri*, and (**c**) *P. kaakan* under RCP scenarios. Maps were generated by ArcMap 10.8.1(https://desktop.arcgis.com/en/arcmap/index.html).

The spatial distribution of habitat suitability showed that for fish *A. latus*, three areas with high suitability including the northwest of the Persian Gulf, the south of the China Sea, and the west of the Philippine Sea were discernible under the four RCPs (Fig. 4a). The extent of these areas begins to decrease from RCP 2.6 to RCP 8.5 (Fig. 4a). For *P. klunzingeri*, the areas with high suitability included the northwest of the Persian Gulf, and around the Strait of Hormuz in the Persian Gulf which showed the increasing trend of the size of high suitable areas towards RCP 8.5 (Fig. 4b). For *A. latus*, a very low range of environments with high suitability was observed in the Strait of Hormuz and west of the Persian Gulf, and the largest extent of the areas with high suitability belonged to RCP 6.0 (Fig. 4c).

### Observed records and habitat preferences of commercial fish

**Figure 5:**
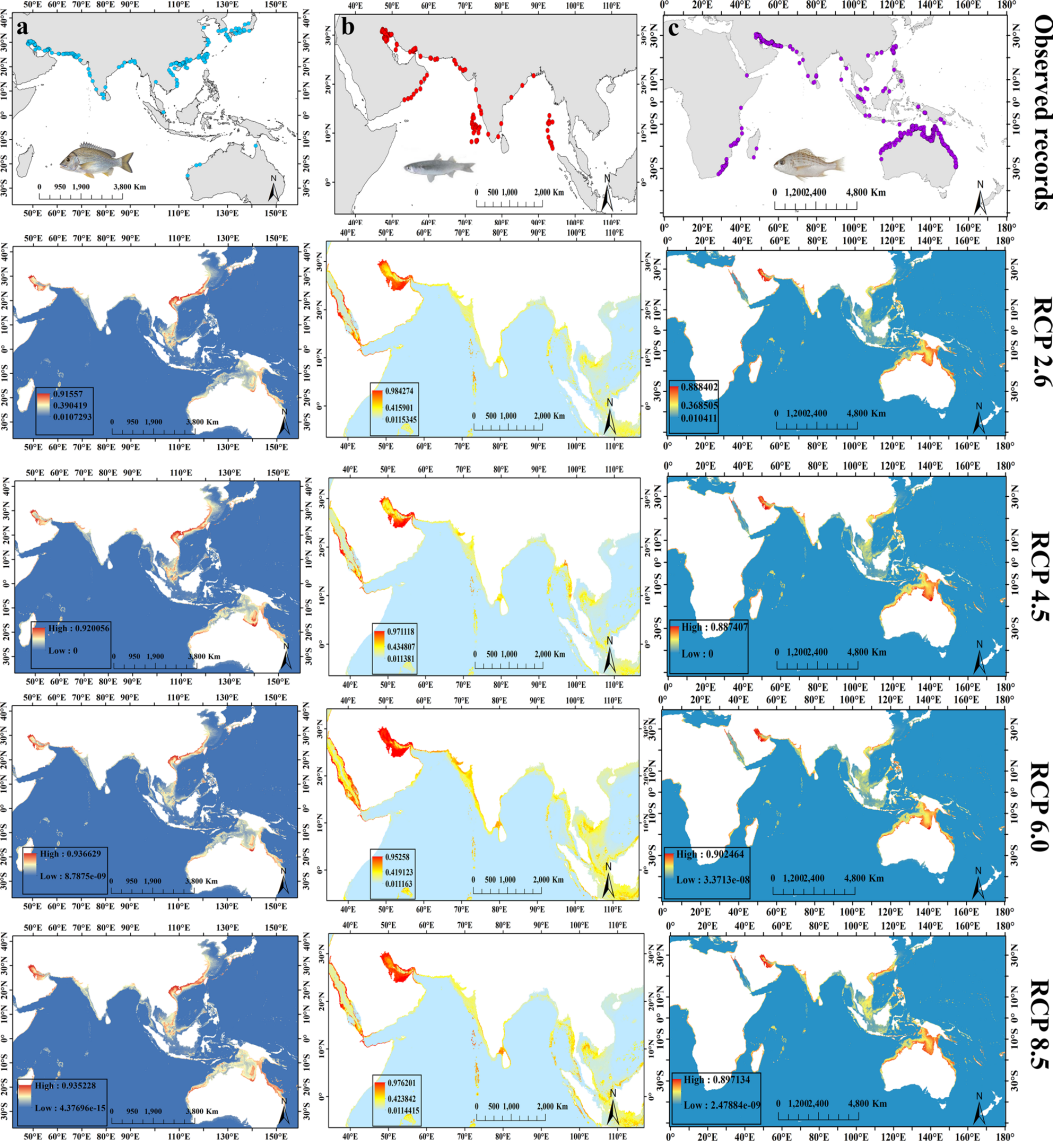
The distribution maps of commercial fish showing observed records (Colored dots) of species, and predicted distribution (Red color indicates the highest occurrence probability and habitat suitability for species) in the future model for three species (**a**) *A. latus*, (**b**) *P. klunzingeri* and (**c**) *P. kaakan* under different scenarios. Maps were generated by ArcMap 10.8.1(https://desktop.arcgis.com/en/arcmap/index.html).

According to observed distributional records of fishes, species *A. latus* and *P. kaakan* showed global distribution in the Persian Gulf, the Oman Sea, and the Indian and Western Pacific Oceans (Fig. 5a, c). For *P. klunzingeri*, records limited to the Persian Gulf, the Oman Sea, the East, and West Indian Ocean, and the Bay of Bengal were visible (Fig. 5b). According to the RCP scenarios, the habitat preferences of the *A. latus* in the future would be the Persian Gulf, the South and East China Sea, the Northwest Philippine Sea, and the northern coast of Australia (Fig. 5a). The Persian Gulf, the Red Sea, and the Lakshadweep tropical archipelago in southern India would be habitat preferences of *P. klunzingeri* under the future scenarios (Fig. 5b). Finally for *P. kaakan,* habitat preferences were included the Persian Gulf and the northern coast of Australia under the future RCPs (Fig. 5c).

**Figure 6:**
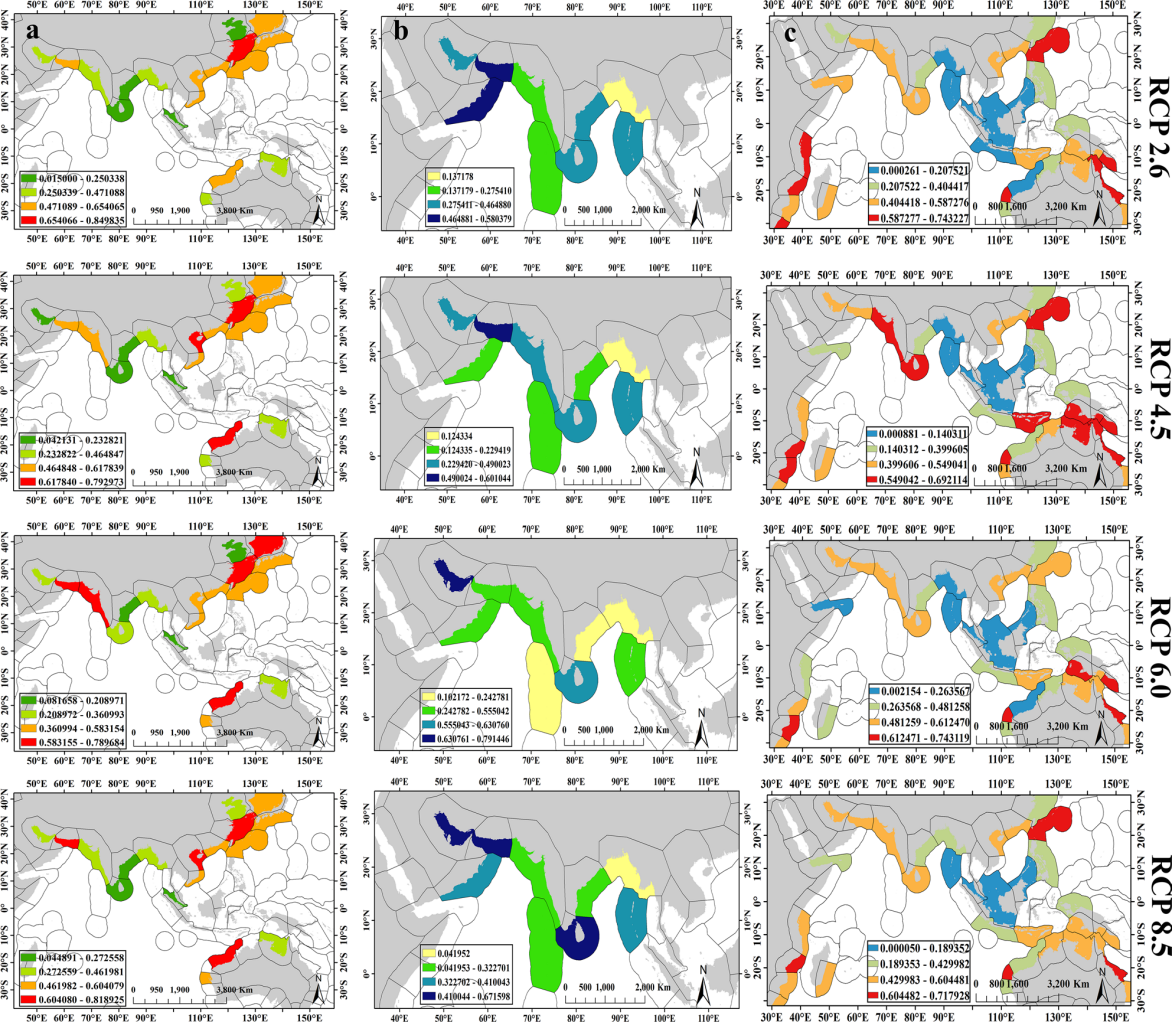
Eco-regions presenting the present and future projections of habitat suitability for three species (**a**) *A. latus*, (**b**) *P. klunzingeri*, and (**c**) *P. kaakan*. Higher values show the higher probability of species occurrence in eco-region. Maps were generated by ArcMap 10.8.1(https://desktop.arcgis.com/en/arcmap/index.html).

In present model, eco-regions Gulf of Oman, Gulf of Tonkin, and East China Sea are most suitable environments for *A. latus* (Fig. 6a). Under climate changes in future, species will prefer environments at higher latitudes including Sea of Japan and Exmouth to Broome (Northwest of Australia) (Fig. 6a). For fish *P. klunzingeri*, eco-regions with high suitability are Gulf of Oman and Maldives in present model (Fig. 6b). It seems that species *P. klunzingeri* will move towards the adjacent areas of eco-regions Gulf of Oman and Maldives including the Persian Gulf and South India and Srilanka following future climate changes (Fig. 6b). Six eco-regions with high suitability including Gulf of Oman, Western India, Southern China, Papua, Bonaparte coast, and Arnhem coast to Gulf of Carpenteria (The last two eco-regions include the northern coasts of Australia) were recognizable for *P. kaakan* in present model (Fig. 6c). Under future scenarios, the distribution of fish *P. kaakan* would be expand towards higher latitudes and eco-regions South Kuroshio (Adjacent to Southern China) in Northern hemisphere, and Bight of Sofala/Swamp Coast (The eastern coast of Africa in Mozambique), Central and Southern great barrier reef and Ningaloo (Western and Eastern coasts of Australia) in Southern hemisphere would be habitat preferences of this species (Fig. 6c).

## Discussion

Our findings support that climate changes will probably affect commercial fish through changes in habitat preferences. A gradual poleward distribution expansion was predicted for commercial fish *A. latus* and *P. kaakan* across RCP scenarios. We observed a decreasing trend of the environment with high suitability towards the future for *P. kaakan* (17% in the present compared to 2% in the future model). Our projections suggest variability of the probability of occurrence of the species is higher in habitats of high suitability compared to environments with moderate or low suitability over the scenarios. Changes in the probability of occurrence were strong in regional scales on eco-regions which are probably due to changes in the optimal environmental conditions of commercial fish. These results are consistent with the findings provided by Lima et al., (2022)^53^, Silva et al., (2019)^42^, and (2016)^52^ on pelagic fish which predict a decrease in habitat suitability under future scenarios.

Our future model of all climatic scenarios predicted that potential preference areas of commercial fish are located in depths below 70 m. It was also observed low variability of optima temperature and salinity among scenarios. It seems the environmental optima of commercial fish be species-specific so that habitat suitability will decrease above or below this environmental interactive range. Our projections suggested temperature probably plays the main role in shaping and distributional variability of commercial fish across future scenarios. Many aspects of the organisms’ biology and ecology are affected by increased temperature^54^. Local conditions will probably determine the final direction of the consequences of increased temperature on marine organisms^55,56^. The habitat preferences of our studied species in the present model were mainly subtropical areas^32^. We observed the reduction of suitable habitats in these areas for studied fish under future scenarios. The overview of previous reports indicated that tropical and subtropical zones will be the most affected by increased temperature^57–59^ so that it was observed the negative effects on the physiology of fish^37^, a drop of up to 40% in the capture potential in marine fisheries^59^, reducing landings^60^, and shorter fishing periods^58^. Moreover, the preferred depth of the species can show their sensitivity to climate change^61^. The studied fish may have a higher sensitivity to temperature increase since benthic species have physiologically adapted to constant temperatures under the surface layers and even small temperature changes in the future may have negative effects on these fish^61–64^.

Following temperature, salinity was the strongest environmental predictor of the distribution of fish *P. klunzingeri*. Jghab et al., (2019)^65^ reported the indirect influence of salinity on sardine distribution while salinity may also be a climate-driven factor inducing shifts in environmental optima^53^. The strong relationship between temperature and salinity in *P. klunzingeri* indicates the role of salinity on species distribution is probably through its effect on temperature. We observed low variations of current velocity among different scenarios as reported for commercial shrimps and fish^66^ and Europen sardine^53^. Moreover, an overview of current velocity and depth showed a higher dependency of three species on deeper and more turbulent waters towards RCP 8.5 by 2100. Abrupt warming can affect stable deeper regions less than superficial waters so that species would probably adapt successfully to the conditions of these regions in the future^5,10,67,68^.

We observed specific responses of commercial fish to ocean warming. Fish *A. latus* and *P. kaakan* showed higher sensitivity to climate change with distributional changes towards poles, while *P. klunzingeri* had moved to nearby habitats. Our study species as highly consumed commercial fish have high regional exploitation rates, especially in the Persian Gulf^18,20,21,25^ resulting in overfishing stocks. Overfishing may aggravate the threats of climate change on stocks of marine species^54,69^. It is suggested the reduction of fishing intensity on highly suitable habitats for studied commercial fish where fishing hotspots are expected. Moreover, small-scale fisheries may face the greatest impact of global warming (Especially for species with high distribution changes and moving towards the poles such as *A. latus* and *P. kaakan*) since expensive or technologically complex adaptations would be required in their present state^54,70^.

## Conclusions

We projected habitat preferences and distribution changes in commercial fish *A. latus*, *P. klunzingeri*, and* P. kaakan* for the first time. The use of a large number of species occurrence records in this study provided high modeling performance in predicting changes in the actual distribution of these commercial fish across future scenarios. Among the four investigated environmental variables, temperature had a significant role in the shaping of the distribution patterns and showing the habitat preferences of these commercial fish. However, the small number of investigated environmental predictors can increase the relative contribution of temperature in predicting the distribution of these commercial fish. The results revealed the sensitivity to climate change is significantly different between the species. Our modeling findings predicted the shrinking of the suitable habitats for these commercial fish, especially in the fish *P. kaakan.* The findings provided in this study including distribution changes across future scenarios, the percentage of suitable and available habitats, and habitat preferences of these commercial fish can be used as basis data to support and manage these habitats for suitable exploitation of commercial fish stocks. To deal with the inevitable threats of climate change on commercial fish, the precautionary principle suggests human uses and fishing activities should be limited in highly suitable habitats of commercial fish. The use of a wider range of commercial fish and multiple environmental variables, as well as modeling at a regional scale, will help to make a more accurate prediction of the habitat preferences of commercial fish in the future.

## Methods

### Occurrence records of species

Online databases GBIF, OBIS, and literature were used to extract observed records of the geographical distribution of commercial fishes including *A. latus*, *P. klunzingeri*, and *P. kaakan* from Sep to Dec 2022. We extracted the records available in the literature showing fishing landing in the Persian Gulf, Oman Sea, and other sites worldwide. Distribution data of GBIF and OBIS were overlapped to avoid the duplication of records^71–74^ and finally, the total dataset was cleaned (where geographical records were on land, or where records had no geographic coordinates)^75,76^ through ArcMap 10.8.1^77^. Finally, we extracted 1531 geographical records of three species from OBIS (829 records, 54%), GBIF (17 records, 1%), and literature (685 records, 45%).

### Future environmental data

We used the database Bio-ORACEL (Marine data layers for ecological modeling) to extract benthic layers with a minimum depth of environmental variables including temperature °C, salinity PSS, and currents velocity m^-1^ for a future period (2090–2100) under four RCPs including RCP 2.6, RCP 4.5, RCP 6.0, and RCP 8.5 in the resolution of 5 arc-min^78,79^. The Global Marine Environmental Datasets (GMED) were used to extract the depth layer at a spatial resolution of 5 arc-min^80^ since it was not available in Bio-ORACEL.

### Setting of MaxEnt

MaxEnt 3.4.1e was selected to model the future distribution of commercial fish^81^. We used MaxEnt since it performs well when used as a habitat suitability index, and shows high predictive performance even with small sample sizes^82,83^. The geographical records of three species (including 371, 171, and 989 records for *A. latus*, *P. klunzingeri*, and *P. kaakan,* respectively) were converted to a single dataset (1531 records) and imported to MaxEnt. Environmental layers of each RCP were separately imported to MaxEnt. The output format of layers in MaxEnt was set to “Logistic” and file type “asc”. The importance of environmental predictors was measured through the “jackknife” option. The “Response curve” option was used to assess the relationship between environmental variables and the predicted presence probability of species. The dataset of records was divided into 75% for training and 25% for testing. We configured the maximum number of iterations to 1000 as suggested by Basher and Costello (2016)^84^ and Saeedi et al. (2016)^73^. The random background points were set at 100,000, and the run type “cross-validate” with 10 replicates was selected. We selected the option “Remove duplicate presence records” to avoid duplicate observations within individual pixels of background environmental layers.

### Output interpretation of MaxEnt

The outputs were separately saved for each RCP. Interpretation of outputs was performed by file “MaxEnt Results”. Habitat suitability was interpreted by “logistic model output” (File type: asc). This output shows the presence probability of species, with values defined from 0 to 1, where 0 means no probability of species presence and unsuitable habitat, middle values suggest medium probability of presence and medium suitability of habitat, and 1 indicates the highest probability of presence and high suitability of habitat^81,85,86^. The Minimum Presence Threshold (MPT) (Showing the minimum probability of species presence) was used to classify the presence probability of species into four classes including the values below MPT (Not Suitable; NS), MPT to 0.5 (Low Suitability; LS), 0.5–0.75 (Medium Suitability; MS), and 0.75–1 (High Suitability, HS)^85^. The performance of MaxEnt was evaluated by the area under the receiver operating characteristic (ROC) curve (AUC)^81^ so that values of AUC above 0.9 indicate the great performance of MaxEnt^87^. The relative importance of environmental variables in predicting the future distribution of commercial fish was assessed through outputs “the percent variable contribution” and “jack-knife” in MaxEnt.

### Analysis data

Violin plots of habitat suitability, temperature, and salinity were plotted by https://www.bioinformatics.com.cn/en (A free online platform for data analysis and visualization). The non-linear polynomial curve of five orders was used to assess the relationship between temperature and salinity in latitude 5°. ArcMap 10.8.1 was used to map habitat suitability^77^. We used Shapefile “Marine Eco-regions of the World (MEOW)”^88^ to map habitat suitability within eco-regions. The tool “Spatial join” in ArcMap was used to extract values of habitat suitability in eco-regions. Habitat suitability was classified (unsuitable to high suitability) through the tool “IDW” (Inverse distance weighted) in ArcMap.

### Supplementary Information

Supplementary Figures.

## Data Availability

Supporting materials are available as Appendix S1 in Supporting Information.

## References

[1] Holsman KK, Haynie AC, Hollowed AB (2020). Ecosystem-based fisheries management forestalls climate-driven collapse. Nat. Commun..

[2] Pecl GT, Araújo MB, Bell JD, Blanchard J (2017). Biodiversity redistribution under climate change: Impacts on ecosystems and human well-being. Science.

[3] Poloczanska ES, Burrows MT, Brown CJ (2016). Responses of marine organisms to climate change across oceans. Front. Mar. Sci..

[4] Gattuso J-P, Magnan A, Billé R (2015). Contrasting futures for ocean and society from different anthropogenic CO_2_ emissions scenarios. Science.

[5] Ramos Martins M, Assis J, Abecasis D (2021). Biologically meaningful distribution models highlight the benefits of the Paris Agreement for demersal fishing targets in the North Atlantic Ocean. Glob. Ecol. Biogeogr..

[6] Pinsky ML, Selden RL, Kitchel ZJ (2020). Climate-driven shifts in marine species ranges: Scaling from organisms to communities. Ann. Rev. Mar. Sci..

[7] IPCC. IPCC Special Report on The Ocean and Cryosphere in a Changing Climate. 10.1007/s13280-019-01313-8 PMC7413947 31994026

[8] Champion C, Hobday AJ, Tracey SR, Pecl GT (2018). Rapid shifts in distribution and high-latitude persistence of oceanographic habitat revealed using citizen science data from a climate change hotspot. Glob. Chang. Biol..

[9] Cheung WWL, Lam VWY, Sarimento JL (2010). Large-scale redistribution of maximum fisheries catch potential in the global ocean under climate change. Glob Chang Biol..

[10] Jones MC, Dye SR, Fernandes JA (2013). Predicting the impact of climate change on threatened species in UK waters. PLoS ONE.

[11] Poloczanska ES, Brown CJ, Sydeman WJ (2013). Global imprint of climate change on marine life. Nat. Clim. Chang..

[12] Kibria G (2013). Climate Change and Agricultural Food Production.

[13] Weatherdon LV, Magnan AK, Rogers AD, Rashid Sumaila U, Cheung WWL (2016). Observed and projected impacts of climate change on marine fisheries, aquaculture, coastal tourism, and human health: An update. Front. Mar. Sci..

[14] Robinson LM, Hobday AJ, Possingham HP, Richardson AJ (2015). Trailing edges projected to move faster than leading edges for large pelagic fish habitats under climate change. Deep Sea Res. Part II Top. Stud. Oceanogr..

[15] Kleisner KM, Fogarty MJ, McGee S (2016). The effects of sub-regional climate velocity on the distribution and spatial extent of marine species assemblages. PLoS ONE.

[16] Melo-Merino SM, Reyes-Bonilla H, Lira-Noriega A (2020). Ecological niche models and species distribution models in marine environments: A literature review and spatial analysis of evidence. Ecol. Modell..

[17] Diaz-Carballido PL, Mendoza-González G, Yañez-Arenas CA, Chiappa-Carrara X (2022). Evaluation of shifts in the potential future distributions of carcharhinid sharks under different climate change scenarios. Front. Mar. Sci..

[18] Parsa M, Yousef Paighambari S, Kamrani E, Nekuru A (2017). CPUE, CPUA and biomass of *Pomadasys kaakan* in the waters of Bushehr province (Persian Gulf). J. Mar. Sci. Technol..

[19] Tang G, He Z, Liu Y (2023). *Acanthopagrus latus* migration patterns and habitat use in Wanshan Islands, Pearl River Estuary, determined using otolith microchemical analysis. Front. Mar. Sci..

[20] Vahabnezhad A, Taghavimotlagh SA, Ghodrati Shojaei M (2017). Growth pattern and reproductive biology of *Acanthopagrus latus* from the Persian Gulf. J. Surv. Fish. Sci..

[21] Panahibazaz M, Taghavi Motlagh SA, Fatemi SMR, Kaymaram F, Vosoghi G (2012). Growth parameter and mortality estimates of yellowfin seabream *Acanthopagrus latus* (Houttuyn, 1782) in the coastal waters of Hormozgan Province, Iran. JOC..

[22] Bastami KD, Afkhami M, Mohammadizadeh M (2015). Bioaccumulation and ecological risk assessment of heavy metals in the sediments and mullet Liza klunzingeri in the northern part of the Persian Gulf. Mar. Pollut. Bull..

[23] Mohammadizadeh M, Afkhami M, Bastami K, Ehsanpour M, Khazaali A, Soltani F (2012). Determination of some biochemical values in the blood of Liza klunzingeri from the coastal water of the Persian Gulf. Afr. J. Biotechnol..

[24] Kohkan O, Abdi R, Zorrieh Zahra SJ, Movahedinia A (2016). Histopathological study of maid fish (*Liza klunzingeri*) in Bandar Abbas Coast line suspected to Viral Nervous Necrosis. Vet. Res. Biol. Prod..

[25] Vahabnezhad A, Hashemi SA, Taghavimotlagh SA, Daryanbard G (2021). Length based spawning potential ratio (LBSPR) of javelin grunter, *Pomadasys kaakan* (Cuvier, 1830) in the Persian Gulf and Oman Sea. Iran. J. Fish. Sci..

[26] Swartz W, Sumaila R, Watson R (2013). Global ex-vessel fish price database revisited: A new approach for estimating ‘missing’ prices. Environ. Resour. Econ..

[27] FAO. *The State of World Fisheries and Aquaculture*. (2022). 10.4060/cc0461en

[28] Sharifinia M, Daliri M, Kamrani E (2019). Estuaries and coastal zones in the Northern Persian Gulf (Iran). Coasts and Estuaries.

[29] Hosseini, S., Daliri. M., Raeisi, H., Paighambari, S., Kamrani, E. Destructive effects of small-scale shrimp trawl fisheries on by-catch fish assemblage in Hormozgan coastal waters. *J. Fish. (in Persian)*. (2014).

[30] Daliri M, Kamrani E, Paighambari SY (2015). Illegal shrimp fishing in Hormozgan inshore waters of the Persian Gulf. Egypt. J. Aquat. Res..

[31] Daliri M, Kamrani E, Jentoft S, Paighambari SY (2016). Why is illegal fishing occurring in the Persian Gulf? A case study from the Hormozgan province of Iran. Ocean Coast Manag..

[32] Sharifian S, Mortazavi MS, Nozar SLM (2023). Predicting present spatial distribution and habitat preferences of commercial fishes using a maximum entropy approach. Environ. Sci. Pollut. Res..

[33] Chen Y, Shan X, Ovando D, Yang T, Dai F, Jin X (2021). Predicting current and future global distribution of black rockfish (*Sebastes schlegelii*) under changing climate. Ecol. Indic..

[34] IPCC. *Climate Change 2014 – Impacts, Adaptation and Vulnerability: Part A: Global and Sectoral Aspects: Working Group II Contribution to the IPCC Fifth Assessment Report: Volume 1: Global and Sectoral Aspects* Vol 1 (Cambridge University Press, 2014). 10.1017/CBO9781107415379

[35] Lam VWY, Sumaila UR, Dyck A, Pauly D, Watson R (2011). Construction and first applications of a global cost of fishing database. ICES J. Mar. Sci..

[36] Sumaila UR, Lam V, Le Manach F, Swartz W, Pauly D (2016). Global fisheries subsidies: An updated estimate. Mar. Policy.

[37] Lam VWY, Cheung WWL, Reygondeau G, Sumaila UR (2016). Projected change in global fisheries revenues under climate change. Sci. Rep..

[38] Wabnitz CCC, Lam VWY, Reygondeau G (2018). Climate change impacts on marine biodiversity, fisheries and society in the Arabian Gulf. PLoS ONE.

[39] Vaidyanathan G (2017). Climate change complicates fisheries modeling and management. Proc. Natl. Acad. Sci..

[40] Wishner KF, Seibel BA, Roman C (2018). Ocean deoxygenation and zooplankton: Very small oxygen differences matter. Sci Adv..

[41] Briscoe NJ, Elith J, Salguero-Gómez R (2019). Forecasting species range dynamics with process-explicit models: Matching methods to applications. Ecol. Lett..

[42] Silva C, Leiva F, Lastra J (2019). Predicting the current and future suitable habitat distributions of the anchovy (*Engraulis ringens*) using the Maxent model in the coastal areas off central-northern Chile. Fish. Oceanogr..

[43] Cheung LWW, Frölicher TL (2016). Building confidence in projections of the responses of living marine resources to climate change. ICES J. Mar. Sci..

[44] Hollowed AB, Barange M, Beamish RJ (2013). Projected impacts of climate change on marine fish and fisheries. ICES J. Mar. Sci..

[45] Schickele A, Goberville E, Leroy B (2021). European small pelagic fish distribution under global change scenarios. Fish Fish..

[46] Elith J, Phillips SJ, Hastie T, Dudík M, Chee YE, Yates CJ (2011). A statistical explanation of MaxEnt for ecologists. Divers. Distrib..

[47] Mendoza-González G, Martínez ML, Rojas-Soto O, Téllez-Valdés O, Arias-Del RI (2016). Priority areas for conservation of beach and dune vegetation of the Mexican Atlantic coast. J. Nat. Conserv..

[48] Elith J, Graham CH, Anderson RP (2006). Novel methods improve prediction of species’ distributions from occurrence data. Ecography (Cop)..

[49] Kaky E, Nolan V, Alatawi A, Gilbert F (2020). A comparison between Ensemble and MaxEnt species distribution modelling approaches for conservation: A case study with Egyptian medicinal plants. Ecol. Inf..

[50] Yousefi M, Jouladeh-Roudbar A, Kafash A (2020). Using endemic freshwater fishes as proxies of their ecosystems to identify high priority rivers for conservation under climate change. Ecol. Indic..

[51] Panja S, Podder A, Homechaudhuri S (2021). Modeling the climate change impact on the habitat suitability and potential distribution of an economically important hill stream fish, *Neolissochilus hexagonolepis*, in the Ganges–Brahmaputra basin of Eastern Himalayas. Aquat. Sci..

[52] Silva C, Andrade I, Yáñez E (2016). Predicting habitat suitability and geographic distribution of anchovy (*Engraulis ringens*) due to climate change in the coastal areas off Chile. Prog. Oceanogr..

[53] Lima ARA, Baltazar-Soares M, Garrido S (2022). Forecasting shifts in habitat suitability across the distribution range of a temperate small pelagic fish under different scenarios of climate change. Sci. Total Environ..

[54] Bueno-Pardo J, Nobre D, Monteiro JN (2021). Climate change vulnerability assessment of the main marine commercial fish and invertebrates of Portugal. Sci. Rep..

[55] Weidberg N, Bularz B, López-Rodríguez S, Navarrete SA (2018). Wave-modulation of mussel daily settlement at contrasting rocky shores in central Chile: Topographic regulation of transport mechanisms in the surf zone. Mar. Ecol. Prog. Ser..

[56] Gomes I, Peteiro LG, Albuquerque R (2016). Wandering mussels: Using natural tags to identify connectivity patterns among Marine Protected Areas. Mar. Ecol. Prog. Ser..

[57] Herrera Montiel SA, Coronado-Franco KV, Selvaraj JJ (2019). Predicted changes in the potential distribution of seerfish (*Scomberomorus sierra*) under multiple climate change scenarios in the Colombian Pacific Ocean. Ecol. Inform..

[58] Alabia ID, Saitoh SI, Igarashi H (2016). Future projected impacts of ocean warming to potential squid habitat in western and central North Pacific. ICES J. Mar. Sci..

[59] Cheung WWL, Watson R, Pauly D (2013). Signature of ocean warming in global fisheries catch. Nature.

[60] Mohammed, E. Y. & Uraguchi, Z. B. Impacts of climate change on fisheries: Implications for food security in Sub-Saharan Africa. *Glob Food Secur Nov Sci Publ Inc*. 114–135 (2013).

[61] Brito-Morales I, Schoeman DS, Molinos JG (2020). Climate velocity reveals increasing exposure of deep-ocean biodiversity to future warming. Nat. Clim. Chang..

[62] Ashford OS, Kenny AJ, Barrio Froján CRS (1884). Phylogenetic and functional evidence suggests that deep-ocean ecosystems are highly sensitive to environmental change and direct human disturbance. Proc. R. Soc. B Biol. Sci..

[63] Levin LA, Le Bris N (2015). The deep ocean under climate change. Science (80-)..

[64] Pinsky ML, Eikeset AM, McCauley DJ, Payne JL, Sunday JM (2019). Greater vulnerability to warming of marine versus terrestrial ectotherms. Nature.

[65] Jghab A, Vargas-Yañez M, Reul A (2019). The influence of environmental factors and hydrodynamics on sardine (*Sardina pilchardus*, Walbaum 1792) abundance in the southern Alboran Sea. J. Mar. Syst..

[66] Sharifian S, Mortazavi MS, Mohebbi Nozar SL (2023). The ecological response of commercial fishes and shrimps to climate change: Predicting global distributional shifts under future scenarios. Reg. Environ. Chang..

[67] Dulvy NK, Rogers SI, Jennings S, Stelzenmüller V, Dye SR, Skjoldal HR (2008). Climate change and deepening of the North Sea fish assemblage: A biotic indicator of warming seas. J. Appl. Ecol..

[68] Jorda G, Marbà N, Bennett S, Santana-Garcon J, Agusti S, Duarte CM (2020). Ocean warming compresses the three-dimensional habitat of marine life. Nat. Ecol. Evol..

[69] Ramírez F, Pennino MG, Albo-Puigserver M, Steenbeek J, Bellido JM, Coll M (2021). SOS small pelagics: A safe operating space for small pelagic fish in the western Mediterranean Sea. Sci. Total Environ..

[70] Ojea E, Lester SE, Salgueiro-Otero D (2020). Adaptation of fishing communities to climate-driven shifts in target species. One Earth.

[71] Sharifian S, Kamrani E, Saeedi H (2021). Insights toward the future potential distribution of mangrove crabs in the Persian Gulf and the Sea of Oman. J. Zool. Syst. Evol. Res..

[72] Sharifian S, Kamrani E, Saeedi H (2021). Global future distributions of mangrove crabs in response to climate change. Wetlands..

[73] Saeedi H, Basher Z, Costello MJ (2016). Modelling present and future global distributions of razor clams (Bivalvia: Solenidae). Helgol. Mar. Res..

[74] Sharifian S, Mortazavi MS, Mohebbi-Nozar SL (2022). Modeling present distribution commercial fish and shrimps using MaxEnt. Wetlands..

[75] Robertson DR (2008). Global biogeographical data bases on marine fishes: caveat emptor. Divers. Distrib..

[76] Saeedi H, Reimer JD, Brandt MI (2019). Global marine biodiversity in the context of achieving the Aichi Targets: Ways forward and addressing data gaps. PeerJ.

[77] ESRI. *ArcGIS desktop: Environmental Systems Research Institute*. (2020).

[78] Tyberghein L, Verbruggen H, Pauly K, Troupin C, Mineur F, De Clerck O (2012). Bio-ORACLE: A global environmental dataset for marine species distribution modelling. Glob. Ecol. Biogeogr..

[79] Assis J, Tyberghein L, Bosch S, Verbruggen H, Serrão EA, De Clerck O (2018). Bio-ORACLE v2.0: Extending marine data layers for bioclimatic modelling. Glob. Ecol. Biogeogr..

[80] Basher, Z., Bowden, D. A. & Costello, M. J. Global Marine Environment Datasets (GMED). World Wide Web electronic publication. Version 2.0 (Rev.02.2018). Accessed at http://gmed.auckland.ac.nz on Access DATE.

[81] Phillips SJ, Anderson RP, Schapire RE (2006). Maximum entropy modeling of species geographic distributions. Ecol. Modell..

[82] Rhoden CM, Peterman WE, Taylor CA (2017). Maxent-directed field surveys identify new populations of narrowly endemic habitat specialists. PeerJ..

[83] Latif QS, Saab VA, Mellen-Mclean K, Dudley JG (2015). Evaluating habitat suitability models for nesting white-headed woodpeckers in unburned forest. J. Wildl. Manag..

[84] Basher Z, Costello MJ (2016). The past, present and future distribution of a deep-sea shrimp in the Southern Ocean. PeerJ..

[85] Phillips SJ, Dudík M (2008). Modeling of species distributions with Maxent: New extensions and a comprehensive evaluation. Ecography (Cop)..

[86] Phillips SJ, Anderson RP, Dudík M, Schapire RE, Blair ME (2017). Opening the black box: An open-source release of Maxent. Ecography (Cop)..

[87] Peterson A, Soberón J, Pearson R (2011). Ecological Niches and Geographic Distributions (MPB-49).

[88] Spalding MD, Fox HE, Allen GR (2007). Marine ecoregions of the world: A bioregionalization of coastal and shelf areas. Bioscience.

