# Supplementary material for: Projected habitat preferences of commercial fish under different scenarios of climate change
Source: Sci Rep. 2024 May 3;14:10177. doi: 10.1038/s41598-024-61008-3 (PMC11068754; doi:10.1038/s41598-024-61008-3)
Supplement: Supplementary file 1 — Supplementary Figures. [file 41598_2024_61008_MOESM1_ESM.docx]

**Projected habitat preferences of commercial fish under different scenarios of climate change**

Sana Sharifian, Mohammad Seddiq Mortazavi, Seyedeh Laili Mohebbi Nozar


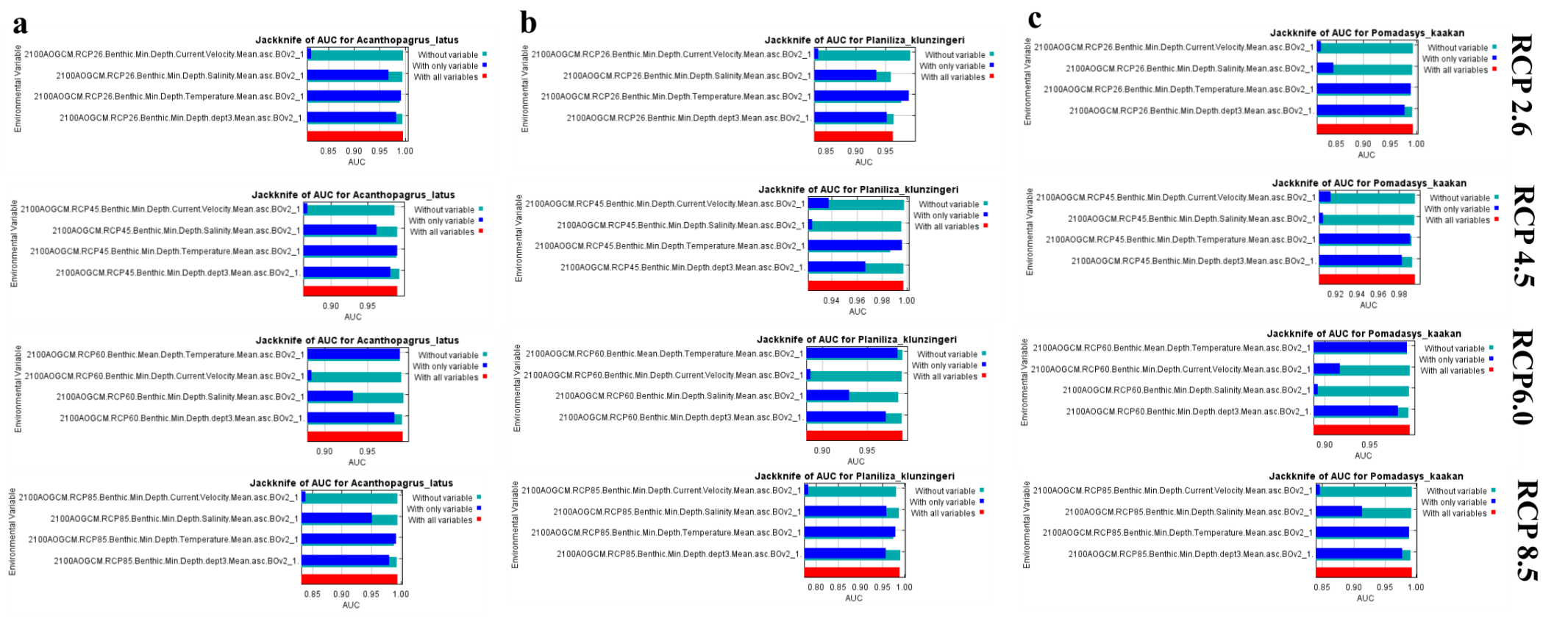


**Figure s1.** The output of "jack-knife" showing relative importance of environmental predictors in three species A) *A. latus*, B) *P. klunzingeri* and C) *P. kaakan* under RCP scenarios.


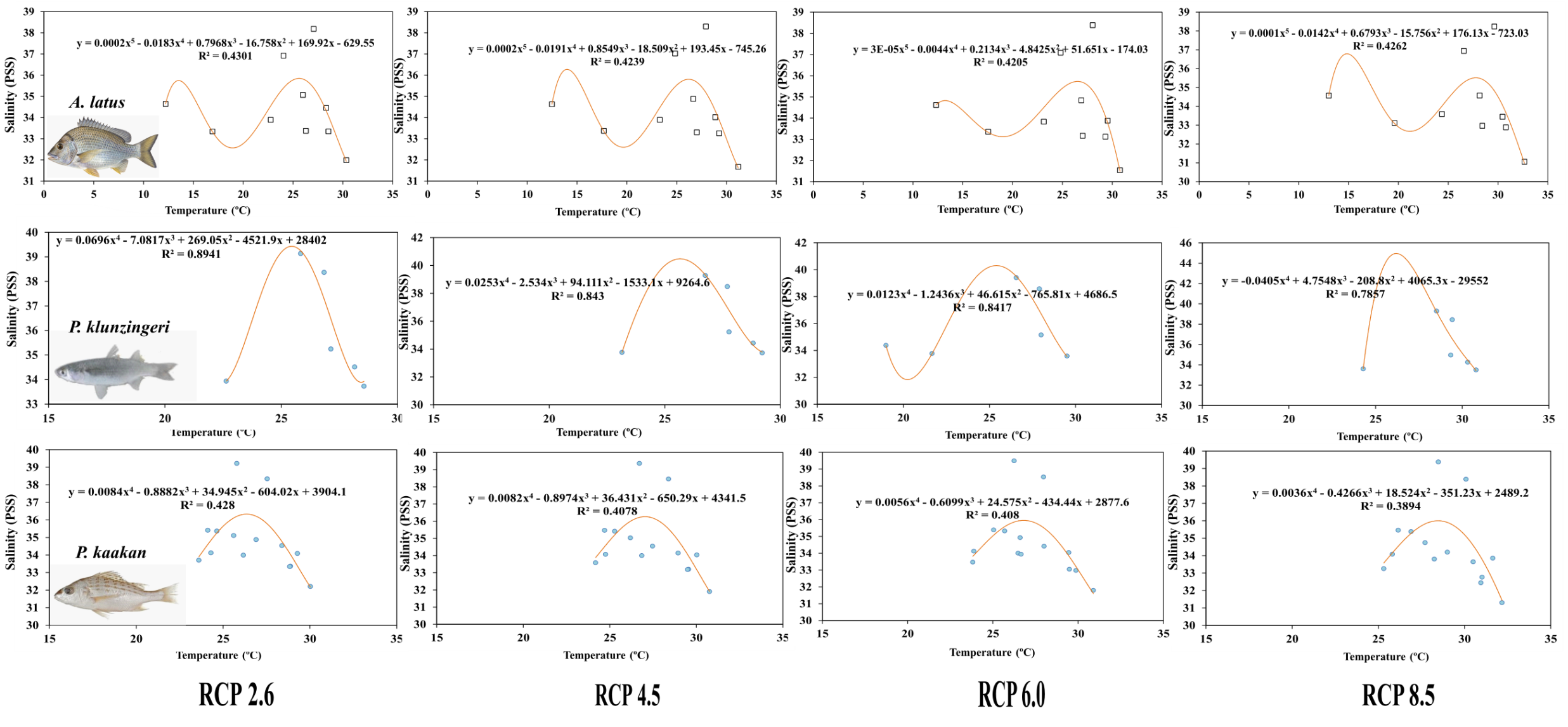
**Figure s2.** The polynomial curves with five polynomial orders showing the relation between temperature and salinity in latitude 5◦ in three species under different scenarios of climate change.
